# Tapering-pressure VAC therapy for wound exudation in POPF after pancreatoduodenectomy: a single-center experience

**DOI:** 10.3389/fsurg.2025.1612420

**Published:** 2025-07-10

**Authors:** Guo-Hua Liu, Zhen-Yue Xu, Jian-Hui Tan, Jia-xing Li, Jun-Er Xu, Xiao-Yu Tan, Jing-Wei Zhai, Jia-yuan Wu, Guo-Hui Zhong, Ming-Yi Li

**Affiliations:** ^1^ Institute of Surgery, Jinan University, Guangzhou, Guangdong, China; ^2^ Department of Hepatobiliary Surgery, Affiliated Hospital, Guangdong Medical University, Zhanjiang, Guangdong, China

**Keywords:** postoperative pancreatic fistulas, open pancreaticoduodenectomy, laparoscopic pancreaticoduodenectomy, vacuum-assisted closure, wound exudation

## Abstract

**Background:**

Pancreaticoduodenectomy(PD) is the only effective treatment for the peri-ampullar carcinoma. However, postoperative pancreatic fistula(POPF) is the most intractable complication causing relevant mortality. Moreover, pancreatic juice may exude from the wound that would lead to more serious complications. Tapering pressure of wall vacuum-assisted closure (VAC) therapy is considered one of the best treatment to wound exudation. Here, we report on a single center series of 5 POPF cases accompanying wound exudation following open or Laparoscopic-assisted pancreatoduodenectomy, successfully managed by VAC.

**Methods:**

We enrolled all patients who experienced POPF ensuing wound exudation following open or Laparoscopic-assisted pancreatoduodenectomy (OPD or LAPD) and received tapering pressure of vacuum-assisted closure (VAC) therapy between July 2017 and August 2024. For VAC, we utilized wall suction device devised by our center applying the technique of negative pressure wound therapy(NPWT). And we adjusted the tapering pressure of the abdominal wound wall vacuum which fixed to a 8Fr or 12Fr suction catheter and connected to the pressure regulator between −50 and −100 mmHg according to the wound exudation amount. When the amount of the wound exudation were less than 100 ml, the wall vacuum suction catheter could be connected to the negative pressure balloon so that the patients could be able to get out of bed. The wall vacuum of VAC was removed when the pancreatic fistula had sufficiently healed which resulting in complete wound healing.

**Results:**

A total of 60 patients underwent OPD or LAPD. Among them, 9 had occured clinically related pancreatic fistulaI(CR-POPF)according to International Study Group on Pancreatic Fistula grade (POPF; 30%). one of the 3 grade C patients underwent Re-laparotomy due to the completely separated pancreaticojejunostomy and postoperative hemorrhage. 5 of the 6 grade B patients was performed tapering pressure of wall vacuum-assisted closure therapy for pancreatic juice exudation from the wound, and all of these patients had good outcomes by this VAC therapy.

**Conclusion:**

Tapering pressure of wall VAC therapy could be a safe and effective treatment in the management of POPF ensuing wound exudation following open or Laparoscopic-assisted pancreatoduodenectomy. And this therapy may potentially reduce POPF-associated mortality.

## 1 Introduction

Pancreaticoduodenectomy (PD) is a complex and technically challenging surgical procedure used for the effective treatment of benign and malignant periampullary lesions, including peri-ampullary carcinoma, neoplastic or preneoplastic disorders of the pancreatic head, distal bile duct, and major duodenal papilla (1–3). In recent years, with the development of minimally invasive surgery, pancreatic minimally invasive centers have gradually shifted from open to laparoscopic-assisted PD, and finally to completely laparoscopic PD. However, postoperative pancreatic fistula (POPF) remains a challenging issue that needs to be addressed in the minimally invasive transformation of PD. Specifically, both OPD and LAPD require an upper abdominal incision, which increases the risk of pancreatic juice exudation from the wound (WEPJ)when POPF occurs. Understanding the differences in WEPJ incidence between these approaches is critical for optimizing perioperative strategies during the adoption of LAPD.

POPF is the most common and severe complication leading to patient mortality after PD surgery (4–6), with an incidence ranging between 5% to 30% in various studies (7, 8). It often occurs due to poor healing or separation of the pancreas-gastric or pancreas-intestinal anastomosis, resulting in leakage of pancreatic juice. POPF can lead to increased morbidity, prolonged hospital stays, and in severe cases, mortality arising from associated complications such as abdominal infections or hemorrhage (9). Among them, the most difficult scenario is POPF caused by separation of the pancreas-gastric or pancreas-intestinal anastomosis, as the volume of leaked pancreatic fluid is often large and difficult to contain (10, 11). For patients who have undergone open or laparoscopic-assisted PD, a significant amount of pancreatic juice can exude from the wound, leading to wound infection, tissue necrosis, and impaired wound healing. If not properly managed, it can result in serious consequences such as intra-abdominal bleeding, sepsis, and even death (12). Currently, various approaches are used to manage POPF caused by separation of the pancreas-gastric or pancreas-intestinal anastomosis, including debridement, drainage, pancreaticoenteric or pancreas-gastric reconstruction, and even total pancreatectomy (13, 14). However, these methods may not be suitable for cases where POPF is accompanied by wound exudation of pancreatic fluid.

Therefore, it is imperative for surgeons to explore more effective strategies for managing such complications, aiming to reduce patient suffering and improve patient outcomes. In recent years, vacuum-assisted closure (VAC) therapy has emerged as a promising technique for managing wound exudation resulting from POPF. This method employs negative pressure to promote wound healing by enhancing blood flow, reducing edema, and facilitating the formation of granulation tissue (15). The application of VAC therapy has shown positive outcomes in various clinical scenarios, including chronic wounds and surgical site infections, yet its specific utility in the context of POPF following PD is still under investigation (16). Although there have been case reports on the application of Vacuum-Assisted Closure (VAC) therapy for traumatic pancreatic injuries resulting in postoperative wound leakage of pancreatic fluid, and the use of endovascular treatment (EVT) for POPF caused by pancreas-gastric anastomotic separation (13, 17), there are no reported studies on the application of VAC therapy for the treatment of wound leakage of pancreatic fluid following PD surgery.

Hence, the purpose of this study is to report a pilot series of patients who developed POPF after PD, followed by wound leakage of pancreatic fluid, and subsequently received Tapering pressure of wall VAC therapy. Overall, understanding and addressing the challenges associated with POPF and wound exudation after PD surgery is crucial for improving patient care and outcomes. This study aims to fill the gap in the existing literature by evaluating the effectiveness of tapering pressure VAC therapy in managing POPF-associated wound exudation following open or laparoscopic-assisted PD. Over a period from July 2017 to August 2024, patients with clinically relevant POPF who received this novel management approach will be analyzed. By employing a wall suction device integrated with a negative pressure regulator, the study will assess the impact of adjusted tapering pressures on wound healing and patient outcomes.

## 2 Methods

### 2.1 Statement of ethics

**Figure 1:**
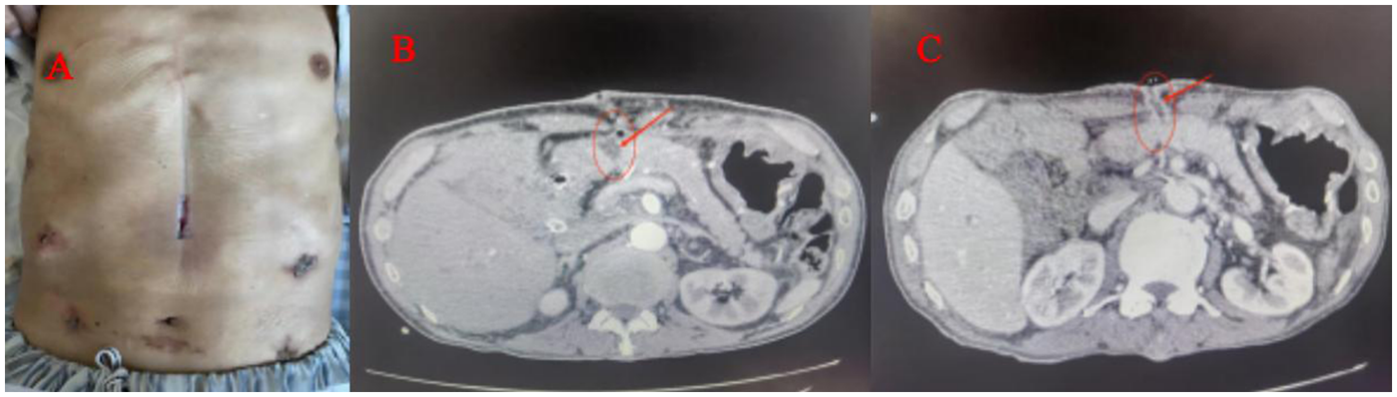
**(A)** POPF ensuing wound exudation pancreatic juice, which caused the skin around the wound redness and hot pain. **(B)** Contrast-enhanced CT shows fluid collections and a gap (arrows)of pancreaticojejunostomy. **(C)** When the amount of the wound exudation were less than 100 ml, repeat CT enhancement show that the effusion was reduced and a fistula (arrows) formed between the pancreatic anastomosis and the incision in the abdominal wall.

**Figure 2:**
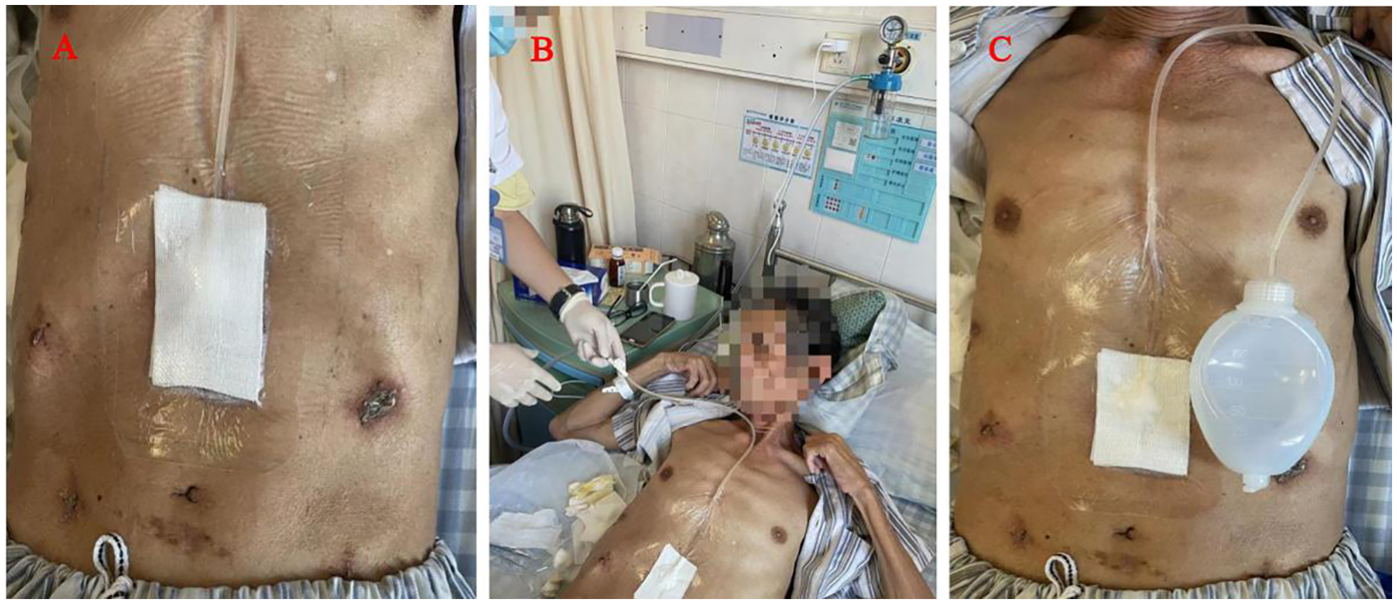
(Patient consent obtained): **(A)** A simple VAC suction device **(B)** the VAC suction device connected to the pressure regulator next to the hospital bed. **(C)** The wall vacuum suction catheter could be connected to the negative pressure balloon, When the amount of the wound exudation were less than 100 ml.

The protocol of this study were approved by the Ethics Committee of Affiliated Hospital of Guangdong Medical University(Approval number: PJKT2022-036), While retrospective data usage was granted a waiver, specific written consent was obtained for the publication of clinical images in Figures 1, 2, with all identifiable features removed.

### 2.2 Patients and data collection

#### 2.2.1 Study design

Retrospective cohort study of consecutive patients undergoing OPD/LAPD (July 2017–August 2024).

#### 2.2.2 Inclusion

All periampullary tumor patients receiving PD.

#### 2.2.3 Exclusion

Emergency conversions, total pancreatectomies, or incomplete records

**Figure 3:**
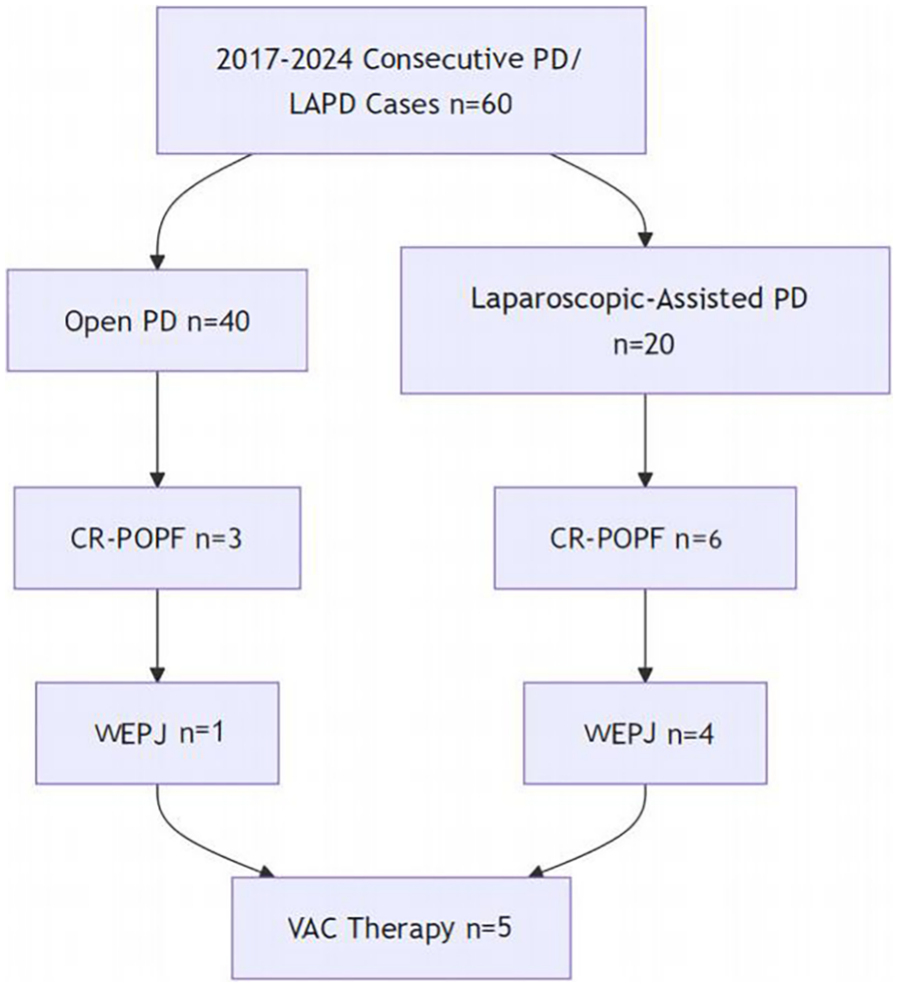
Flowchart of consecutive OPD/LAPD cases: enrollment, complications, and VAC therapy.

From July 2017 to August 2024, All patients who underwent OPD or LAPD for periampullary tumors at Guangdong Medical University Hospital were evaluated. Of the 60 patients, 40 underwent OPD and 20 underwent LAPD. The perioperative data were collected, including, the patients’ backgrounds and preoperative characteristics (sex; age; body mass index; pancreatic texture; pancreatic duct diameter; histopathological diagnosis and Fistula Risk Score (FRS) which was calculated per Callery et al. (18), incorporating pancreatic texture, duct size, pathology, and intraoperative blood loss; the way of pancreaticojejunostomy; and the postoperative complications such as the rate of clinically related pancreatic fistula(CR-POPF), postoperative hemorrhage(PH), Delayed gastric emptying(DGE), Disrupted pancreaticojejunostomy(DPJ) and Peripancreatic fluid accumulation (PFA), etc. All consecutive PD/LAPD cases during the study period were included without selection. The enrollment pathway is summarized in Figure 3.

### 2.3 Operative procedure

All patients who underwent OPD or LAPD were conducted using a uniform surgical technique by a consistent surgical team. Currently, numerous studies have identified the method of pancreaticojejunostomy as one of the significant factors influencing postoperative pancreatic fistula (19–21). To minimize bias due to technical differences, all cases utilized the modified Blumgart pancreaticojejunostomy technique (21), which had good outcomes by the preliminary research of our surgical team.

### 2.4 Diagnosis of the main postoperative complications

The definition and grading of POPF was based on the 2016 ISGPF classification (22). The definitions of DGE and PH were based on the ISGPS (23, 24). The diagnosis of disrupted PJ was based on the symptoms in patients presenting with sepsis and bleeding, elevated drain fluid amylase levels, and radiographic findings on contrast-enhanced CT (11).

### 2.5 Postoperative management after OPD or LAPD

**Figure 4:**
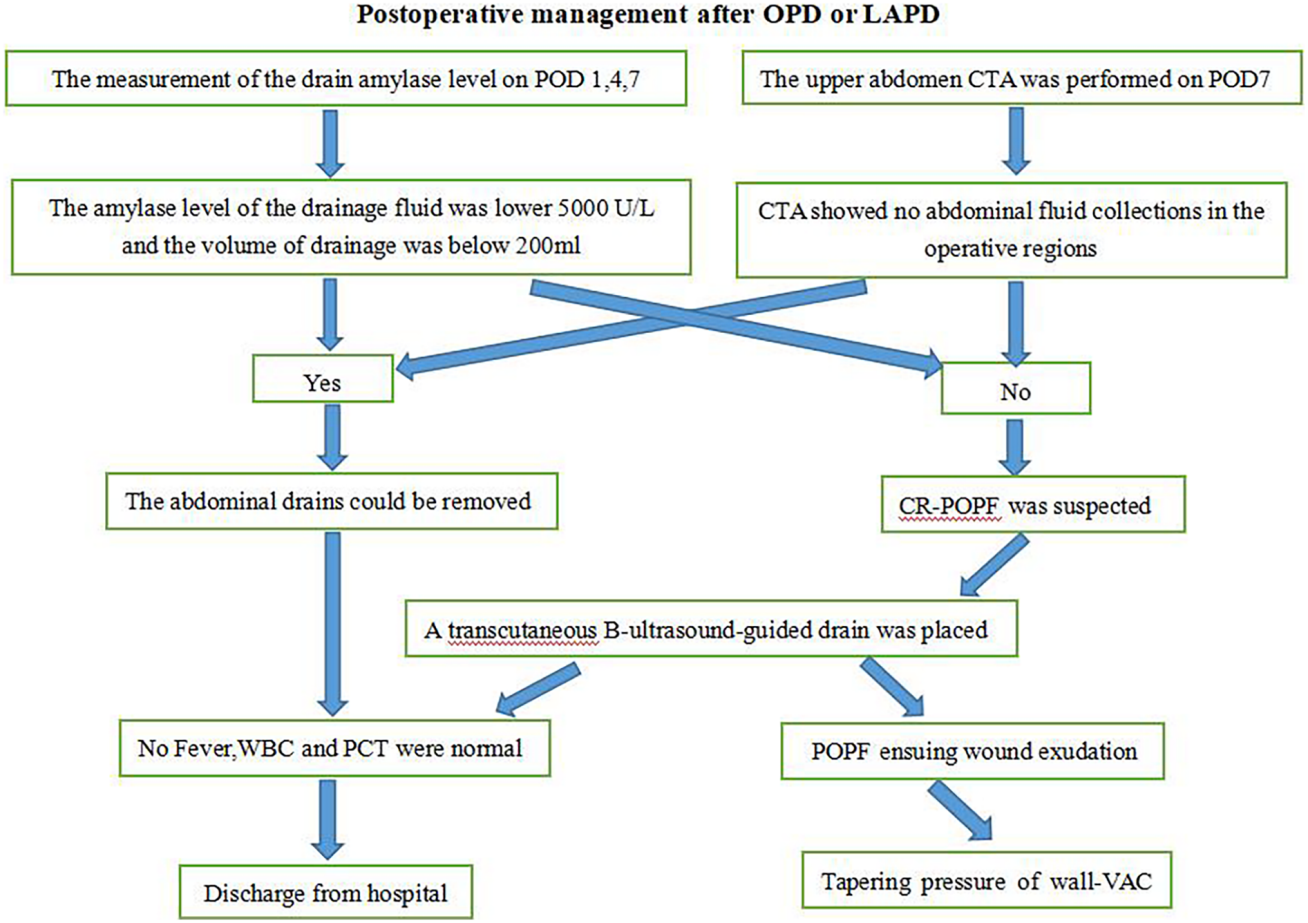
Procedure for the management of pancreatic fistula. OPD, open pancreatoduodenectomy; LAPD, laparoscopic-assisted pancreatoduodenectomy; POD, post operation day; CTA, computed tomography angiography; POPF, postoperative pancreatic fistula; CR-POPF, clinically related pancreatic fistulaI; WBC, white blood cells; PCT, procalcitonin.

All patients who had been performed surgical procedure for OPD or LAPD were implemented the standard postoperative management. Three drains were placed at the pancreaticojejunostomy (PJ) site, hepatocolic ligament, and Morrison's pouch after surgery. And the volume and amylase of the drain fluid were routinely measured on POD 1, 4, and 7, enhanced CT of the upper abdomen was routinely performed between POD 5 and POD 7. and the drains were removed if there was no evidence of POPF if amylase levels of the drain fluid were <3× serum on POD 7 and CT enhancement indicates no fluid accumulation in the abdominal cavity or around the anastomotic site. Pancreatic duct stents were used. Perioperative antibiotics (cefoperazone/sulbactam) were administered for 48 h, and somatostatin analogs (octreotide 0.1 mg subcutaneously every 8 h) were continued until POD 5. Pancreatic texture (soft/hard) was intraoperatively assessed by palpation, and pancreatic duct diameter was measured via preoperative MRI and MRCP. But if CR-POPF was suspected, Patients should be treated according to the different conditions. In case of abdominal fluid collections which could be located by B ultrasound, transcutaneous guided drain was placed. If POPF ensuing wound exudation had been occurred, tapering pressure of wall vacuum-assisted closure therapy was the treatment of choice for these patients (Figure 4).

### 2.6 Diagnosis and management of POPF ensuing wound exudation

The diagnosis of POPF ensuing wound exudation was based on the symptoms of the fluid flowing out of the wound after OPD or LAPD (Figure 1A), and the fluid amylase levels were higher than 3-fold of the normal blood and contrast-enhanced CT shows fluid collections and a gap (arrows)of pancreaticojejunostomy (Figure 1B).

**Figure 5:**
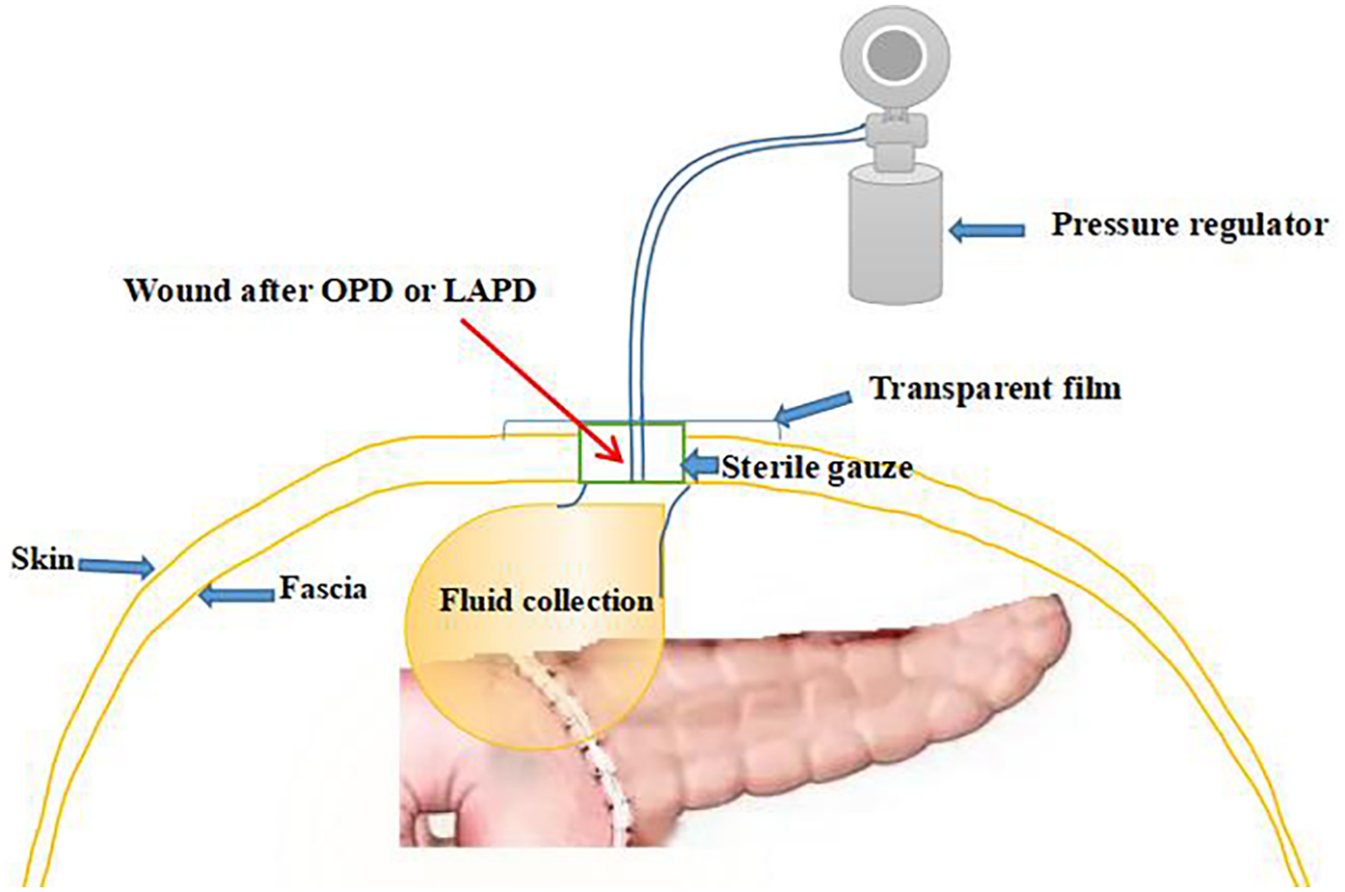
Diagrammatic sketch of VAC suction device installation.

We applied tapering pressure of VAC therapy to treat the above patients, and received a good treatment effect. Here is the specific treatment procedure (Figure 5). (1) We designed a VAC suction device according to the wound of the patients: A sterile gauze was cut into appropriate size around 8 Fr or 12 Fr suction catheter, put it into the wound, then cover the sterile gauze, suction tube from the wound attached to the skin, then paste transparent film so that it would make a vacuum state among the film and skin, drainage tube and the gauze (Figure 2A). (2) The above attraction tube connected to the pressure regulator between −50 and −100 mmHg according to the wound exudation amount (Figure 2B). (3) When the amount of the wound exudation were less than 100 ml, repeat CT enhancement is required. If the effusion was reduced and a fistula formed between the pancreatic anastomosis and the incision in the abdominal wall (Figure 1C), the wall vacuum suction catheter could be connected to the negative pressure balloon so that the patients could be able to get out of bed (Figure 2C). (4) The wall vacuum of VAC was removed when the pancreatic fistula had sufficiently healed which resulting in complete wound healing.

### 2.7 Statistical analyses

Statistical analysis was conducted with SPSS 25.0 software package. The Kruskal–Wallis test was used to compare continuous variables and to interpret nonparametric variables. The Chi-square or Fisher's exact test was used to evaluate frequencies between categorical variables. A value of *p* < 0.05 was considered significant.

## 3 Results

Figure 3 illustrates the flow of 60 consecutive patients undergoing PD/LAPD. Of these, 9 developed CR-POPF, with 5 cases (1 OPD, 4 LAPD) receiving VAC for WEPJ.

### 3.1 Backgrounds and preoperative characteristics

60 patients have been performed OPD or LAPD with the same PJ in our centers since July 2017. Table 1 summarize the preoperative characteristics of these 60 patients. It shows that the tumor site of LAPD's patients was mainly Ampulla of Vater. The average Operation time and blood loss of the LAPD group are no significant differencesthe compared with the OPD group. The same results occurred in the other background characteristics and surgical outcomes between OPD and LAPD involving age, sex, weight, ALT, ALB, TB, hospital stay, pancreatic textur**e**, pancreatic duct diameter and FRS. However, The average BMI and ALB of LAPD are worse significantly than that of OPD.

**Table 1:** Background and preoperative of the patients after OPD or LAPD.

Variable	OPD (*n* = 40)	LAPD (*n* = 20)	t/*x*^2^	*P*-value
Sex, *n* (%)			0.136	0.713
Male	18 (45%)	8 (40%)		
Female	22 (55%)	12 (60%)		
Age (years)	57.3 ± 9.3	57.5 ± 8.6	−0.070	0.944
Weight (kg)	59.3 ± 5.7	56.9 ± 7.2	1.424	0.160
BMI (kg/m^2^)	22.3 ± 2.1	19.6 ± 2.6	4.221	<0.001
Hepatic function index				
ALT	56.0 ± 12.8	59.2 ± 15.2	−0.871	0.387
ALB	33.0 ± 2.4	31.6 ± 2.1	2.245	0.029
TB	134.2 ± 58.4	128.9 ± 48.0	0.347	0.730
Preoperative biliary drainage			0.170	0.680
Yes	30 (75%)	14 (70%)		
No	10 (25%)	6 (30%)		
Pancreatic duct diameter(mm)			0.330	0.566
≤3	15 (37.5%)	6 (30%)		
>3	25 (62.5%)	14 (70%)		
Pancreatic texture (*n* %)			0.897	0.344
Soft	13 (32.5%)	9 (45%)		
Hard	27 (67.5%)	11 (55%)		
Primary site (*n* %)			0.631	1.000
Pancreas	10 (25%)	5 (25%)		
Bile duct	4 (10%)	1 (5%)		
Ampulla of vater	23 (57.5%)	12 (60%)		
Duodenum	3 (7.5%)	2 (10%)		
Operative characteristics				
Operation time (min)	262.0 ± 28.4	258.0 ± 36.7	0.469	0.641
Blood loss (ml)	125.3 ± 57.1	146.3 ± 75.2	−1.209	0.231
FRS			3.258	0.346
Negligible (0)	2 (5%)	1 (5%)		
Low risk (1–2)	10 (25%)	4 (20%)		
Intermediate risk (3–6)	22 (55%)	8 (40%)		
High risk (7–10)	6(15%)	7(35%)		

BMI, body mass index; ALT, alanineaminotransferase; ALB, albumin; TB, total bilirubin; PJ, pancreaticojejunostomy; FRS, fistula risk score.

### 3.2 Postoperative complications

Table 2 shows that no matter OPD group or LAPD group, there was a low incidence rate of postoperative complications which involved DGE, PH, DPJ, CRPOPF, PFA, biliary leakage and Abdominal infection. No cases were found to be dead within 90 days after the surgery. And two cases of LAPD group required re-operation, one case for bleeding and the other had a grade C pancreatic leak due to DPJ. As for pancreatic fluid out from the wound, one case happened in OPD group, but four cases happened in LAPD group, there are simultaneously statistical differences between two groups. And form Table 1 showing that LAPD patients exhibited lower preoperative BMI and albumin levels compared to OPD (19.6 vs. 22.3 kg/m², *p* < 0.001; 31.6 vs. 33.0 g/L, *p* = 0.029). These findings suggest that nutritional status and body composition may influence the technical challenges of LAPD, potentially contributing to its higher incidence of WEPJ (20% vs. 2.5%, *p* = 0.038).

**Table 2:** Postoperative complications of the patients.

Postoperative complications	OPD (*n* = 40)	LAPD (*n* = 20)	*P*-value
Biliary leakage, *n* (%)	0 (0%)	0 (0%)	1.000
DGE, *n* (%)	6 (15%)	3 (15%)	1.000
Abdominal infection, *n* (%)	2 (5%)	0 (0%)	0.548
PH, *n* (%)	0 (0%)	1 (5%)	0.333
DPJ, *n* (%)	0 (0%)	1 (5%)	0.333
PFA, *n* (%)	5 (12.5%)	6 (30%)	0.155
CRPOPF, *n* (%)	3 (7.5%)	6 (30%)	0.344
Grade B POPF	2 (5%)	4 (25%)	0.089
Grade C POPF	1 (2.5%)	2 (10%)	0.255
Pancreatic fluid out from the wound (WEPJ), *n* (%)	1 (2.5%)	4 (20%)	0.038
RE-laparotomy	0 (0%)	1 (5%)	0.333
Mortality, *n* (%)	0 (0%)	0 (0%)	1.000
Hospital stay (days)	16.8 ± 2.5	15.9 ± 3.6	0.322

PH, postoperative hemorrhage; CRPOPF, clinically related postoperative pancreatic fistula; DPJ, disrupted pancreaticojejunostomy; DGE, delayed gastric emptying; PFA, peripancreatic fluid accumulation; Mortality, death with 90 days after surgery.

### 3.3 VAC therapy for the complication of wound exudation pancreatic juice(WEPJ)

All five patients underwent vacuum-assisted closure (VAC) therapy as part of their postoperative management following pancreatic surgery. The essential VAC therapy data are presented in Table 3. The mean age of the patients was 58.6 years (range 47–73), with a male-to-female ratio of 2:3. The preoperative weight and BMI were consistent across the patient cohort, averaging 49.2 kg and 16.7, respectively.Hepatic function indices revealed that the mean alanine aminotransferase (ALT) was 70.6 U/L, albumin (ALB) was 29.6 g/L, and total bilirubin (TB) was 137.4 μmol/L. Preoperative biliary drainage was performed in four out of five patients. The pancreatic duct diameter was ≤3 mm in three patients and >3 mm in two patients. The pancreatic texture was soft in all cases. The primary surgical site was the ampulla of Vater for four patients and the pancreas for one patient. Operative characteristics showed a mean operation time of 288 min and a mean blood loss of 128 ml. All patients underwent laparoscopic-assisted pancreaticoduodenectomy (LAPD), except for one patient who had an open pancreaticoduodenectomy (OPD). No delayed gastric emptying (DGE), abdominal infection, postoperative hemorrhage (PH), or duodenal perforation (DPJ) was observed. The pancreatic fistula grade (POPF) was B in all patients. The mean FRS was 8.6, and all patients had a positive fluid accumulation (PFA). The mean time to WEPJ was 9.6 days. VAC therapy was initiated with a pressure intensity of −50 to −100 mm Hg, and the mean duration of treatment was 5.6 days. The VAC-pressure balloon was applied with a pressure intensity of <−5 mm Hg, and the mean duration of this treatment was 5 days. Importantly, there were no deaths in this patient cohort. The mean hospital stay was 20.4 days.

**Table 3:** Synopsis of basic vacuum-assisted closure (VAC) therapy data.

Variable	Patient-1	Patient-2	Patient-3	Patient-4	Patient-5
Sex	Male	Female	Female	Male	Female
Age	47	63	73	64	55
Weight (Preoperative)	48	46	49	51	48
BMI	16.5	16.1	16.5	17.8	17.6
Hepatic function index					
ALT	68	72	49	76	88
ALB	30	29	31	28	29
TB	248	157	85	43	121
Preoperative biliary drainage	Yes	Yes	No	No	Yes
Pancreatic duct diameter (mm)	≤3	≤3	≤3	>3	>3
Pancreatic texture	Soft	Soft	Soft	Soft	Soft
Primary site	Pancreas	Ampulla of vater	Ampulla of vater	Ampulla of vater	Ampulla of vater
Operative characteristics					
Operation time (min)	252	280	315	278	340
Blood loss (ml)	100	100	150	100	250
Method of PD	OPD	LAPD	LAPD	LAPD	LAPD
DGE	No	No	No	No	No
Abdominal infection	No	No	No	No	No
PH	No	No	No	No	No
DPJ	No	No	No	No	No
POPF grade	B	B	B	B	B
FRS	7	9	8	9	10
PFA	Yes	Yes	Yes	Yes	Yes
WEPJ (Days post PD)	8	7	10	12	9
VAC-pressure regulator					
Pressure intensity (mm Hg)	−50 to −100	−50 to −100	−50 to −100	−50 to −100	−50 to −100
Duration of the treatment (Days)	5	6	5	6	7
VAC—pressure balloon					
Pressure intensity (mm Hg)	<−5	<−5	<−5	<−5	<−5
Duration of the treatment (Days)	3	5	5	4	7
Death	No	No	No	No	No
Hospital stay (days)	16	18	20	22	22

BMI, body mass index; ALT, alanineaminotransferase; ALB, albumin; TB, total bilirubin; FRS, fistula risk score; PDAC, panceatic ductal adenocarcinoma; IPMN, intraductal papillary mucinous neoplasm; WEPJ, wound exudation pancreatic juice; PH, postoperative hemorrhage; DPJ, disrupted pancreaticojejunostomy; POPF, postoperative pancreatic fistula; DPJ, disrupted pancreaticojejunostomy; PFA, peripancreatic fluid accumulation; VAC, vacuum-assisted closure.

## 4 Discussion

Pancreatic fistula is a common and serious complication following pancreaticoduodenectomy (PD), posing significant risks such as peripancreatic fluid collection, severe infections, and bleeding (7, 8). The evolution of PD techniques has entered a minimally invasive phase, with most centers transitioning through open, assisted, laparoscopic, and robotic approaches. However, during the open and assisted phases, the presence of an upper abdominal incision adds the complication of wound exudation of pancreatic juice (WEPJ), which can lead to poor wound healing and infection. POPF and WEPJ not only complicate the postoperative course but also correlates with increased morbidity and mortality, making its prevention and management a critical focus in pancreatic surgery. In our center, we have summarized the process of transitioning to minimally invasive PD and developed strategies to manage common complications associated with pancreatic fistulas.

In this study, we retrospectively analyzed the perioperative data of patients undergoing open (OPD) and laparoscopic-assisted (LAPD). We found that the tumor site in the LAPD group was predominantly the ampulla of Vater, suggesting that during the transition to minimally invasive PD, surgeons may opt to start with relatively easier cases. Despite this, there were no significant differences between the LAPD and OPD groups in terms of operation time, blood loss, hospital stay, and postoperative complications such as delayed gastric emptying (DGE), postoperative hemorrhage (PH), and clinically relevant postoperative pancreatic fistula (CRPOPF). This indicates that with appropriate case selection, the quality of LAPD is comparable to OPD even in the early stages of technological development.Notably, the incidence of WEPJ as a result of pancreatic fistula was higher in the LAPD group (4 cases) compared to the OPD group (1 case). Analysis of preoperative data revealed that the LAPD group had lower average BMI and albumin (ALB) levels, suggesting that poor nutritional status may be one of the factors contributing to the higher incidence of this complication in the LAPD group. POPF-related wound exudation (WEPJ) and Surgical Site Infections (SSI) share overlapping risk factors, such as prolonged wound exposure and compromised tissue integrity. However, WEPJ is primarily driven by enzymatic leakage of pancreatic fluid, leading to localized inflammation and tissue necrosis, whereas SSI involves bacterial colonization. Distinguishing between the two requires clinical correlation: WEPJ typically presents with high amylase levels in exudate (>3× serum), while SSI is confirmed by purulent discharge, systemic signs of infection (e.g., fever, leukocytosis), and positive microbial cultures (Bassi et al., 2017) (22). In our cohort, all WEPJ cases underwent amylase testing and CT imaging to exclude SSI. All five patients with WEPJ underwent vacuum-assisted closure (VAC) therapy as part of their postoperative management. The VAC therapy data presented in Table 3 show that the mean time to WEPJ was 9.6 days, and the mean duration of VAC therapy was 5.6 days. The application of VAC therapy with a pressure intensity of −50 to −100 mm Hg was effective, with no mortalities observed in this cohort. The mean hospital stay was extended to 20.4 days, highlighting the impact of WEPJ on patient recovery and healthcare costs.

In Addition, the main purpose of our study is to evaluate the efficacy of tapering pressure vacuum-assisted closure (VAC) therapy in managing POPF accompanied by wound exudation following open or laparoscopic pancreatoduodenectomy. We analyzed the patients experiencing clinically relevant POPF (CR-POPF), with a particular focus on those exhibiting pancreatic juice exudation from surgical wounds. Our findings demonstrate that the approach utilizing tapering pressure wall vacuum-assisted closure (VAC) therapy can be a safe and effective intervention, significantly improving wound healing and potentially reducing the morbidity associated with POPF, which highlighting its potential as a valuable intervention in the postoperative management of patients experiencing POPF. The implications of these findings for clinical practice are substantial. The successful application of tapering pressure VAC therapy in managing POPF-related wound exudation suggests that this technique could be incorporated into standard postoperative care protocols for pancreaticoduodenectomy patients. By reducing the incidence of wound complications and promoting faster recovery, VAC therapy may improve overall patient outcomes and reduce hospital stays, which is particularly crucial given the high costs associated with prolonged hospitalization following complex surgical procedures (15). Moreover, as the healthcare community continues to strive for enhanced patient safety and quality of care, integrating VAC therapy into clinical pathways may offer a practical solution to address the complications arising from POPF.

Besides, the higher incidence of WEPJ in LAPD patients may reflect the technical learning curve and patient selection during early adoption of minimally invasive PD. Lower BMI and albumin levels in this group highlight the need for preoperative nutritional optimization and meticulous anastomotic techniques to mitigate POPF risks. These insights are critical for surgeons transitioning from OPD to LAPD, as they underscore the importance of tailoring perioperative management to patient-specific factors. Recent studies have demonstrated the efficacy of VAC therapy in traumatic pancreatic fistulas (Handaya et al.) (17) and postoperative anastomotic leaks (Kaczmarek et al.) (13). However, these reports primarily focused on open or endoscopic approaches, leaving a gap in evidence for LAPD-specific wound complications. In our cohort, the successful application of VAC in LAPD patients (100% healing rate without mortality) not only validates its cross-procedural applicability but also underscores its unique value during the minimally invasive transition—by controlling enzymatic exudation, VAC mitigates secondary infection risks and avoids re-laparotomy (Garnier et al.) (14). Furthermore, the lower BMI and albumin levels in LAPD patients (19.6 vs. 22.3 kg/m², *p* < 0.001) reinforce the importance of preoperative nutritional optimization, complementing Sugimoto et al.'s (2017) (19) ’soft pancreas, small duct’ risk model. Together, these insights provide a multidimensional framework for refining perioperative strategies in LAPD.

However, this study is not without limitations. The limitations of this study primarily stem from its single-center design, which may restrict the generalizability of the findings to broader populations. Additionally, the relatively small sample size of patients undergoing vacuum-assisted closure (VAC) therapy for postoperative pancreatic fistula (POPF) limits the statistical power to draw definitive conclusions regarding the efficacy of this treatment modality. Furthermore, the lack of a control group diminishes the ability to compare outcomes with standard management strategies, potentially introducing bias in assessing the effectiveness of VAC therapy.

## 5 Conclusion

In conclusion, while minimally invasive PD techniques offer promising outcomes, careful patient selection and preparedness for managing complications such as pancreatic fistulas and WEPJ are essential. Our experience and the results of this study contribute to the growing body of knowledge on the perioperative management of PD and the role of VAC therapy in the treatment of WEPJ. In the absence of a control group, tapering pressure wall VAC therapy demonstrates significant potential as a safe and effective intervention for managing POPF associated with wound exudation following open or laparoscopic-assisted pancreatoduodenectomy. This approach not only facilitates a favorable healing environment but also may contribute to reducing morbidity and mortality linked to these complications. While all VAC-treated patients (*n* = 5) achieved healing, the small cohort precludes definitive efficacy conclusions.Multicenter randomized controlled trials(RCTs) are needed to validate tapering-pressure VAC in POPF management.

## Data Availability

The original contributions presented in the study are included in the article/Supplementary Material, further inquiries can be directed to the corresponding author.
